# SOCIAL PRESCRIBING FOR THE ARTS: LESSONS FROM ABROAD FOR THE HEALTH CARE OF OLDER AMERICANS

**DOI:** 10.1093/geroni/igad104.1991

**Published:** 2023-12-21

**Authors:** Shayna Gleason, Sudha Shreeniwas, Joy Birabwa

**Affiliations:** University of Massachusetts, Boston Gerontology, Boston, Massachusetts, United States; University of North Carolina Greensboro, Greensboro, North Carolina, United States; University of North Carolina at Greensboro, Greensboro, North Carolina, United States

## Abstract

Evidence shows the dramatically positive influence of participation in the arts on the health and well-being of older adults (OA). Social Prescribing for the Arts (also called Arts on Prescription or AoP) — in which a professional in a clinical or community setting refers a client to the arts and supports them in accessing arts programming — constitutes one mechanism by which OA may participate in health-promoting arts activities. AoP has been incorporated in other high-income nations including Canada, the UK, and some EU nations. The UK especially has embedded AoP into its healthcare system; however, its universal health insurance program makes its context substantially different from the United States’. This study examined models of AoP abroad and how they could be adapted to the U.S. context for OA. We conducted 25 semi-structured, in-depth interviews with professionals involved in AoP overseas, including physicians, link workers, arts providers, policy experts, and others, as well as 10 interviews with their U.S. counterparts (N=35). Participants were recruited through referral sampling and we intentionally cultivated diversity of role in AoP schemes during recruitment. Transcripts were coded thematically by three team members. Resulting themes included challenges abroad with funding for arts, uneven training among referrers, strain on referrers and arts providers, difficulty reaching the most disadvantaged, skepticism from various stakeholders, the importance of patient involvement in developing a care plan, and a need for open communication between arts providers and referrers. Recommendations for the U.S. include designing and evaluating AoP programs that address these concerns.

